# Aquaporin 3 maintains the stemness of CD133+ hepatocellular carcinoma cells by activating STAT3

**DOI:** 10.1038/s41419-019-1712-0

**Published:** 2019-06-13

**Authors:** Yawei Wang, Gang Wu, Xueyan Fu, Shaolin Xu, Tianlong Wang, Qi Zhang, Ye Yang

**Affiliations:** 1 Department of Geriatric Surgery, The First Affiliated Hospital of China Medical University, Shenyang, PR China; 2 Department of Hepatobiliary Surgery, The First Affiliated Hospital of China Medical University, Shenyang, PR China

**Keywords:** Cancer stem cells, Outcomes research

## Abstract

An increasing interest in liver cancer stemness arises owing to its aggressive behavior and poor prognosis. CD133, a widely known liver cancer stem cell marker, plays critical roles in the maintenance of liver cancer stemness. Thus, exploring the regulatory mechanism of CD133 expression is significant. In the present study, we proved the carcinogenesis roles of aquaporin 3 (AQP3) in hepatocellular carcinoma (HCC) and demonstrated that AQP3 promotes the stem cell-like properties of hepatoma cells by regulating CD133 expression. In addition, AQP3 promoted the stimulation and nuclear translocation of signal transducer and activator of transcription 3 (STAT3) with a subsequent increase in the level of CD133 promoter-acetylated histone H3. This phenomenon accelerated CD133 transcription. Next, whether AQP3 acted as an oncogenic gene in HCC and maintained the stemness of CD133+ hepatoma cells were elucidated; also, a novel mechanism underlying the AQP3/STAT3/CD133 pathway in HCC was deduced.

## Introduction

In China, hepatocellular carcinoma (HCC) is the fourth most commonly diagnosed and the third most prevalent cancer leading to an estimated 422,100 deaths annually^1^. In recent decades, surgical resection is the first-line treatment for HCC; however, it is not applicable to the majority of the advanced HCCs^2^. The long-time survival is unsatisfactory due to the high rate of recurrence and metastasis. Liver cancer stem cells (LCSCs) are highly tumorigenic, metastatic, chemotherapy- and radiation-resistant, and responsible for tumor relapse after therapy^3–5^. Therefore, understanding the mechanism underlying the maintenance of LCSCs is crucial for future treatment strategies.

Aquaporin 3 (AQP3) belongs to aquaporins, a family of water channel proteins found in the plasma membranes of various cells^6^. Recent studies have shown that the aberrant expression of AQP3 contributes to the progression and metastasis of several malignant tumors^7–10^. Accumulating pieces of evidence support that AQP3 is related to the maintenance of stemness not only in normal stem cells but also in cancer stem cells (CSCs)^11–13^. Another study in HCC cells indicated that AQP3 promotes cellular proliferation and invasion^14^. However, the AQP3 expression in HCC patients, the independent influence on prognosis, and the carcinogenic mechanism in HCC progression are yet to be clarified.

In the current study, we found AQP3 was highly expressed in HCC specimens and cell lines. The expression was related to TNM stage, metastasis, and prognosis in HCC. In vitro and in vivo experiments showed that the depletion of AQP3 suppresses the cellular proliferation and invasion. Further investigations revealed that AQP3 promotes the stem cell-like properties of hepatoma cells by regulating the expression of CD133. Interestingly, the janus kinase/signal transducer and activator of transcription 3 (JAK/STAT3) signaling pathway induces the expression of CD133 during liver carcinogenesis^15^, following which, we investigated whether AQP3 could affect the JAK/STAT3 signaling pathway. Results showed that AQP3 activated STAT3 by promoting STAT3 phosphorylation and inducing STAT3 nuclear translocation with a subsequent increase in the levels of acetylated histone H3 of the CD133 promoter. Taken together, we demonstrated that *AQP3* functions as an oncogenic gene in HCC and maintains the stemness of CD133+ hepatoma cells. In addition, we identified a novel mechanism of the AQP3/STAT3/CD133 pathway in HCC.

## Materials and methods

### Patient tissue samples and liver cancer cell lines

HCC tissue slice samples were obtained from 120 patients diagnosed with HCC, who underwent a routine hepatic resection in the First Affiliated Hospital of China Medical University between January 2009 and January 2011. The inclusion criteria of all 120 patients were as follows: the tumor was completely resected without distant organ metastasis and the postoperative pathological diagnosis was HCC. The histological diagnosis and differentiation were evaluated independently by three pathologists using hematoxylin- and eosin-stained slides according to the WHO classification system^16^. None of the patients received preoperative radiotherapy or chemotherapy prior to surgical resection. The follow-up period for survival was 5 years. A total of 37 paired fresh specimens, including tumor tissues and the corresponding paired noncancerous parenchyma, were snap frozen in liquid nitrogen and stored at − 70 °C immediately after resection. The inclusion criteria are the same as above. The project protocol was approved by the Institutional Ethics Committee of China Medical University prior to the initiation of the study. All patients provided informed consent before the study. Liver cancer cell lines Huh7, HCCLM3, SMMC7721, HepG2, Bel7402, PLC/PRF/5, and Hep3B and the normal liver cell line L02 were obtained from the Shanghai Cell Bank (Shanghai, China) and cultured in high-glucose Dulbecco’s-modified Eagle medium supplemented with 10% fetal bovine serum (FBS) and 1% penicillin/streptomycin in a humidified atmosphere containing 5% CO_2_ at 37 °C.

### RNA preparation and quantitative real-time PCR

Total RNA was extracted from ~ 100 mg of the 37 paired tissue samples and liver cancer cell lines using TRIzol (Invitrogen, USA) according to the manufacturer’s instructions. The primers were designed and synthesized by Sangon Biotech Company (Shanghai, China) (Supporting file 1). The *GAPDH* gene was used as an endogenous control. The relative gene expression was assessed using qRT-PCR and expressed by ΔCt = Ct gene−Ct reference; the fold-change in the gene expression was calculated using the 2^−ΔΔCt^ method^17^. Every tissue was assessed three times.

### Western blotting

Total/cytoplasm/nucleus protein was extracted from tumor tissues, non-tumor adjacent tissues, or liver cancer cell lines using the Total/Cytoplasm/Nucleus Protein Extraction Kit (Solarbio, China). An equivalent of 50 µg of the protein extract was resolved by sodium dodecyl sulfate polyacrylamide gel electrophoresis and electrotransferred to polyvinylidene difluoride membranes (Millipore, Billerica, MA, USA). The membrane was blocked for 2 h at room temperature using milk (5%) was used to block membranes. Subsequently, the membranes were probed with primary antibodies, including rabbit polyclonal antibodies to AQP3 (1:2000, Abcam, USA), CD133 (1:1500, Abcam), JAK1 (1:1000, Abcam), pY-JAK1 (1:2000, Abcam), JAK2 (1:1500, Abcam), pY-JAK2 (1:1500, Abcam), STAT3 (1:2000, Abcam), pY^705^-STAT3 (1:2000, Abcam), GAPDH mouse monoclonal antibody (1:2000, Abcam), Histone H3 (phospho S10) rabbit monoclonal antibody (1:500, Abcam) overnight at 4 °C, followed by incubation with secondary antibodies for 2 h at room temperature. The immunoreactive bands were identified using an ECL system (Millipore, USA). Every tissue was evaluated three times using Western blotting.

### Immunofluorescence

HCC cells were seeded in 12-well plates at moderate density and transfected as indicated above. The cells were fixed with 4% paraformaldehyde for 30 min and permeabilized by 1% Triton X-100 in phosphate-buffered saline (PBS) for 20 min at room temperature. After washing in PBS, the cells were incubated with 1% bovine serum albumin (BSA) for 30 min. For immunofluorescence staining, the cells were incubated with AQP3 (1:1000, Abcam) or CD133 antibody (1:1000, Abcam). Then, goat anti-rabbit immunoglobulin G (1:2000, ProteinTech Group, USA) was used as a secondary antibody at 4 °C overnight. Finally, the cells were stained with 4′,6-diamidino-2-phenylindole (Boster, China) to visualize the nuclei, and stained samples were imaged using a fluorescence microscope (Nikon eclipse, Japan). The immunofluorescence assay was conducted three times in each group.

### Immunohistochemistry (IHC)

AQP3/CD133/CD44/CD90/EPCAM expression was analyzed in paraffin-embedded specimens obtained from 120 patients. Four-µm-thick tissue sections were deparaffinized in xylene and dehydrated before antigen retrieval for 5 min using an autoclave. The endogenous peroxidase activity was blocked using hydrogen peroxide (0.3%), and non-specific immunoglobulin binding sites were blocked by normal goat serum for 30 min at 37 °C. Tissue sections were incubated with anti-AQP3 (1:1000, Abcam), anti-CD133 (1:200, Abcam), anti-CD44 (1:1000, Abcam), anti-EPCAM (1:200, Abcam), and anti-CD90 (1:200, Abcam) overnight at 4 °C. Then, the sections were incubated with biotinylated goat anti-rabbit IgG as a secondary antibody (Maixin Kit, China) for 1 h at room temperature, followed by incubation with streptavidin–biotin horseradish peroxidase-conjugated (Maixin Kit) for 30 min at room temperature. The peroxidase reaction was developed with 3′-diaminobenzidine tetrahydrochloride (Maixin Kit). The expression levels of the proteins were scored semi-quantitatively according to the percentage of positively stained cells combined with the staining intensity according to our previous study^18^. The specimens were assessed three times.

### Cell transfection

The HCCLM3 and Huh7 cells were plated in 24-well plates until 60% confluence. Then, the cells were infected with lentivirus-AQP3-shRNAs or lentivirus-AQP3-NC (GenePharma, China) according to the multiplicity of infection values. After 12 h, fresh medium was replaced. Subsequently, the expression of the *GFP* gene was observed under a fluorescence microscope. The effect of the shRNA-lentivirus on the expression of AQP3 was assessed by qRT-PCR. pcDNA-AQP3 was used to upregulated the AQP3 expression, and pcDNA3.1-NC was used as the control. Both plasmids were purchased from GenePharma.

### CCK8 and colony formation assay

Approximately 2000 cells/well were plated in 96-well plates in media containing 10% FBS at 24 h post-transfection. Then, 10 μl CCK8 reagent (Solarbio, China) was added to each well and incubated for an additional 1 h at 37 °C, and the absorbance measured at 450 nm.

After transfection, the cells were harvested in the logarithmic growth phase from the monolayer culture for the colony formation assay with ~ 200 cells/well in six-well plates in media containing 10% FBS. The colony formation was allowed for 2 weeks. Then, the cells were washed with 1 mL PBS, fixed, stained with 0.1% crystal violet solution for 20 min, and finally washed three times with water. The fixed cell colonies were allowed to air dry, and the rate of clone formation was calculated. The CCK8 and colony formation assays were conducted three times in each group.

### Cell cycle analysis

HCCLM3 and HUH7 cells in six-well plates were transfected with Lv-AQP3-shRNAs or Lv-AQP3-NC. After 48 h of transfection, the cells were seeded at a density of 5 × 10^5^/well, trypsinized, fixed with 70% ethanol at 4 °C, and washed with PBS. A volume of 100 μL RNase A was added, and the mixture was incubated in a 37 °C water bath for 30 min. An additional 400 μl propidium iodide (PI; Sigma, USA) staining solution was added and incubated at 4 °C in the dark for 30 min. Then fluorescence-activated cell sorting (FACS) Calibur (Becton Dickinson, USA) was used to detect and record the fluorescence upon excitation at 488 nm. The cell cycle analysis was conducted three times in each group.

### Transwell assay

After transfection, the cells were seeded on the synthetic basement membrane in the inset of a 24-well culture plate. In the invasion assay, polycarbonate filters coated with 50 μl Matrigel (1:9, BD Bioscience, USA) were placed in the transwell chamber (Costar, USA). In the migration assay, no Matrigel was placed in the chambers. FBS was added to the lower chamber of the transwell as a chemoattractant. Then, the cells were incubated at 37 °C to allow invasion through the Matrigel barrier for 24 h. Subsequently, the filters were fixed and stained with 0.1% crystal violet solution. The non-invading cells were removed using a cotton swab, and invading cells on the underside of the filter were enumerated using an inverted microscope (Nikon MicrophotFX, Japan).

### Tumorigenicity experiments in nude mice

A total of 30 male nude mice weighing 18–20 g, provided by Keygen Biotech (Nanjing, China), were bred under aseptic conditions and housed in the presence of constant humidity at 60–70% and room temperature of 18–20 °C. The animal maintenance, husbandry, and experimental procedures were performed in accordance with the rules of China Medical University for the Use of Experimental Animals and approved by the Medical Animal Care and Use Committee of China Medical University (Shenyang, China). Then, the mice were randomly assigned to three groups with HCCLM3 or HUH7 cells injected subcutaneously in the abdomen: Lv-AQP3-NC, Lv-AQP3-shRNA1, Lv-AQP3-shRNA2. Subsequently, the animals were examined every 3 days, and killed at 21 days after the initial injection, followed by resection and weighing of the tumors.

### RT-PCR gene array

Forty-eight hours after *AQP3* knockdown in HCCM3 and HUH7 cells, total RNA was extracted from the cells using TRIzol (Invitrogen) and purified with the RNeasy_MinElute^TM^ Cleanup Kit (Qiagen, Germany). Subsequently, the total RNA was reverse transcribed using Superscript III Reverse Transcriptase (Invitrogen), and the complementary DNA (cDNA) was amplified by PCR using 2_Super Array PCR Master Mix (Qiagen). Then, RT-PCR was performed on each sample using the Human Tumor Proliferation/Invasion RT2 Profiler^TM^ PCR Array (SuperArray Bioscience, USA) in a Thermal Cycler Dice Real-Time System (Takara, Japan) according to the manufacturer’s instructions. The expression data of the target genes were normalized to that of *GAPDH* using the 2^-ΔΔCt^ method.

### FACS

HCCLM3 and HUH7 cells were collected and washed with 1% BSA/cold PBS two times by centrifugation at 1000 rpm for 5 min at 4 °C. CD133 rabbit polyclonal antibody (Abcam) was added to HCCLM3 or HUH7 cells and incubated for 15 min at room temperature in the dark. Then, the cells were washed with PBS, and goat anti-rabbit IgG-labeled FITC (Abcam, USA) was added to the cells for 15 min at room temperature, away from light. Subsequently, the cells were separated, and the proportion of CD133+ cells in HCCLM3 and HUH7 cells was estimated by FACS Calibur.

### Magnetic cell separation

CD133 MicroBeads conjugated to monoclonal anti-human CD133 antibodies were purchased from Miltenyi Biotec (Germany). First, the CD133+ cells in HCCLM3 or HUH7 cells were magnetically labeled with CD133 MicroBeads according to the manufacturer’s instructions. Then, the cell suspension was loaded onto the AutoMACS Pro Separator (Miltenyi Biotec). Consequently, the magnetically labeled CD133+ cells were retained and the unlabeled cells were excluded as the eluate. To increase the purity, the positively selected cell fraction containing the CD133+ cells was re-separated.

### Spheroid formation assay

Sphere formation was performed in ultralow attachment plates (Corning, USA) containing medium supplemented with 2% B27 (Invitrogen), 20 ng/ml basic fibroblast growth factor (PeproTech, USA), and 20 ng/ml human recombinant epidermal growth factor (PeproTech, USA). CD133+ HCCLM3 and CD133+ HUH7 cells were plated at a density of 2 cells/μl and cultured at 37 °C in 5% CO_2_. After 14 days, the spheres > 50 μm diameter were enumerated at × 40 magnification under a microscope (Nikon).

### Side population assay

The CD133+ HCCLM3 and HUH7 cell suspensions were labeled with Hoechst 33342 dye (Molecular Probes, USA) for side population analysis according to the standard protocol^19^. In brief, the cells were resuspended in OPTI-MEM (Gibco, USA) at a density of 10^6^/ml. Hoechst 33342 dye was added at a final concentration of 5 μg/ml in the presence or absence of final concentration 50 μmol/l verapamil, and the cells were incubated at 37 °C for 90 min with intermittent shaking, followed by centrifugation at 1000 r/min at 4 °C for 10 mins. The cell pellet was suspended in chilled OPTI-MEM containing 2% FBS and 10 mmol/L HEPES. In total, 1 μg/ml propidium iodide was added to the cells to gate the viable cells during analysis on a FACS Calibur. The Hoechst 33342 dye was excited at 355 nm.

### Chromatin immunoprecipitation analysis

Chromatin immunoprecipitation (CHIP) was performed using the EpiQuik™ Global Histone H3 Acetylation Assay Kit (Epigentek, USA) according to the manufacturer’s instructions. In brief, cross-linked chromatin DNA was sonicated into 200–1000-bp fragments. This fragmented chromatin was immunoprecipitated using an anti-acetyl-histone H3. Normal mouse IgG was used as the negative control. qRT-PCR was performed to assess the expression of CD133 promoter.

### Statistical analysis

The statistical analyses were performed using the SPSS 19.0 software. Parametric data are presented as mean ± standard error of mean (SEM), and the differences between each group were analyzed using the Student’s *t* test. All the *p* values reported were two-sided, and significance was defined as *p* < 0.05.

## Results

### AQP3 expression in HCC tissues and cell lines

**Fig. 1:**
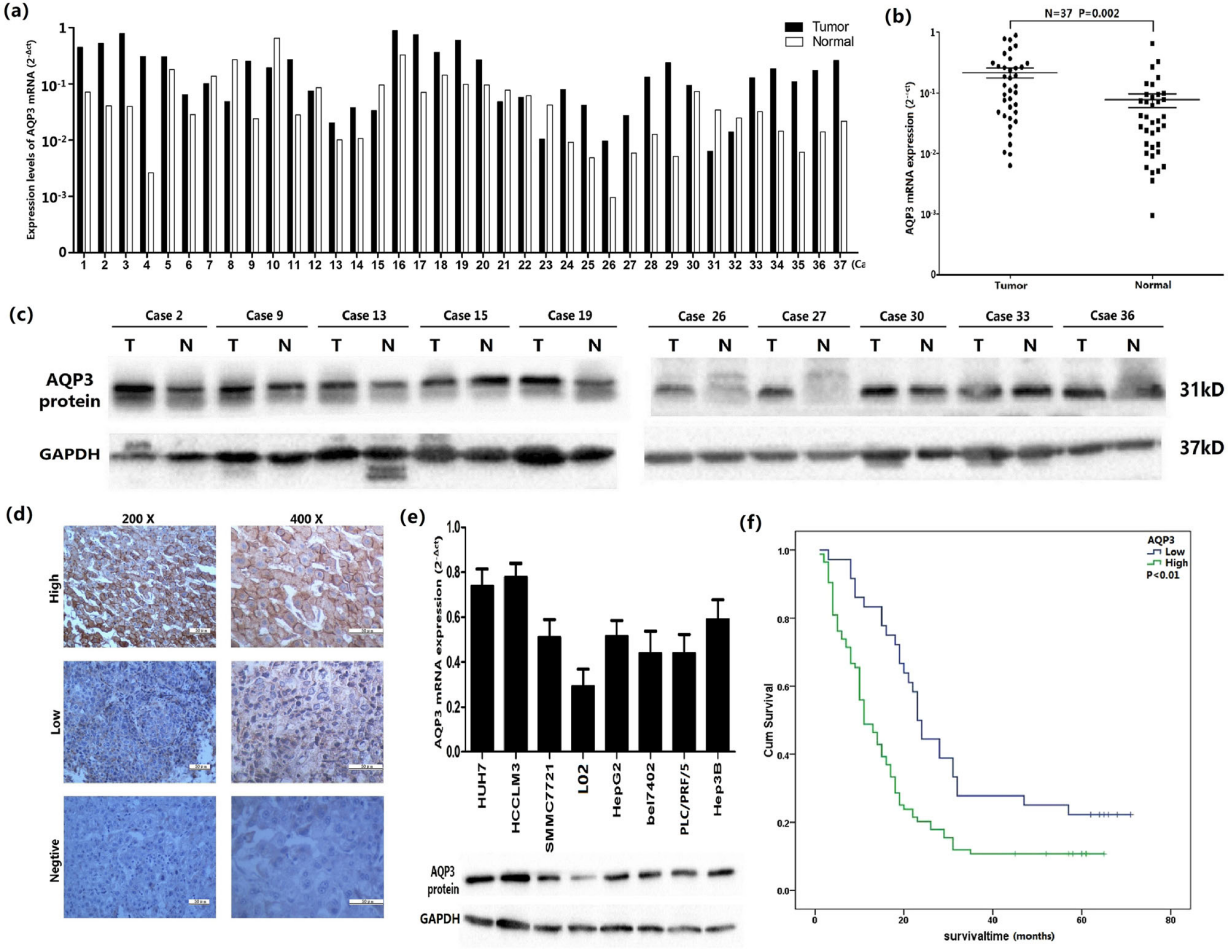
AQP3 expression in HCC samples and cell lines. **a** The expression levels of AQP3 mRNA in 37 cases HCC tumor tissues and the corresponding normal tissues according to qRT-PCR. **b** The average mRNA expression levels (2^−Δct^) of AQP3 in 37 HCC tumor tissues and the corresponding normal tissues according to qRT-PCR. **c** The expression levels of AQP3 protein in 10 cases HCC tumor tissues and the corresponding normal tissues according to western blotting. **d** According to IHC in 120 HCC paraffin section specimens, AQP3 protein appeared to be expressed in the cytomembrane of tumor cells. **e** AQP3 was high expressed in 7 HCC cell lines (HUH7, HCCLM3, SMMC7721, HepG2, bel7402, PLC/PRF/5, Hep3B) than the normal liver cell lines (L02). **f** According to IHC in 120 HCC paraffin section specimens, the overall survival in patients with high AQP3 expression was significantly lower than in patients with low AQP3 expression (*P* < 0.01, KM survival curve)

The expression of AQP3 mRNA was examined in 37 HCC fresh tissue samples using qRT-PCR. Compared with noncancerous tissues, high expression was detected in 27 cases (72.97%) and low in eight cases (21.62%); however, no difference was found among the noncancerous and cancer tissues in two cases (5.41%) (Fig. 1a). Moreover, the mean expression levels of AQP3 mRNA were higher in HCC tissues as compared with the normal tissues (*p* = 0.02, Fig. 1b). Among the 37 fresh tissue samples, 10 were selected randomly to assess the expression of AQP3 protein by western blot. The results showed seven cases with high expression, two with low, and one case with no difference in the expression (Fig. 1c). According to IHC in 120 HCC patients, AQP3 protein was found to be primarily expressed in the cytomembrane of the tumor cells (Fig. 1d). Furthermore, AQP3 was highly expressed in 70% tumor samples (84/120) and low or negatively expressed in the remaining 36 cases (30%) (Table 1). Compared with the normal liver cell line L02, Huh7, and HCCLM3 cells showed maximal AQP3 expression among the seven liver cancer cell lines (Fig. 1e).

**Table 1:** Association between AQP3 expression according to immunohistochemistry and conventional clinicopathological parameters in 120 patients with HCC

Characteristics	Number of patients	AQP3 Low expression	AQP3 High expression	*P*
	120	36	84	
*Age (years)*				
≥ 50	70	21	49	0.582
< 50	50	15	35	
*Gender*				
Male	80	25	55	0.420
Female	40	11	29	
*Tumor size*				
≥ 5 cm	53	18	35	0.260
< 5 cm	67	18	49	
*Metastasis*				
Yes	52	10	42	**0.019**
No	68	26	;42	
*HBsAg status*				
Positive	63	22	41	0.150
Negative	57	14	43	
*Tumor differentiation*				
High	14	3	11	0.721
Moderate	57	17	40	
Poor	49	16	33	
*Cirrhosis*				
Yes	53	15	37	0.485
No	67	21	47	
*Serum AFP*				
< 200 ng/dl	39	13	26	0.364
≥ 200 ng/dl	81	23	58	
*Recurrence*				
Yes	83	26	57	0.402
No	37	10	27	
*TNM stage*				
I + II	55	23	22	**0.040**
III + IV	65	13	52	

Bold fonts indicate statistically significant

### Clinical significance of AQP3 expression in HCC

The IHC results revealed an association between AQP3 expression and various clinicopathological factors in the 120 HCC patients. The AQP3 expression was significantly higher in HCC tissues with advanced TNM stage as compared with those with early TNM stage (*P* = 0.040), and the tissues with AQP3 overexpression exhibited marked tumor metastasis (*p* = 0.019, Table 1). Furthermore, patients with high AQP3 expression showed poorer prognosis. The overall survival was significantly lower in patients with high AQP3 expression than in those with low expression (*p* < 0.01, Fig. 1f). In addition, multivariate analysis demonstrated that AQP3, TNM stage, and α-fetoprotein were significant prognostic factors for HCC patients (Table 2).

**Table 2:** COX regeression regression analysis on the relationship of clinicopathologic characteristics and prognosis

Characteristics	Univariate			Multivariate		
	HR	CI (95%)	*P*	HR	CI (95%)	*P*
Age	0.913	0.621–1.357	0.648			
Gender	1.302	0.867–1.952	0.203			
TNM stage	1.492	1.220–1.826	<**0.001**	1.418	1.150–1.748	**0.001**
Tumor differentiation	1.017	0.761–1.358	0.911			
Metastasis	1.612	1.092–2.379	**0.016**	1.163	0.769–1.758	0.474
Recurrence	1.320	0.862–2.021	**0.201**			
Tumor size	0.822	0.556–1.214	0.324			
Serum AFP	1.843	1.196–2.840	**0.006**	1.803	1.157–2.810	**0.009**
Cirrhosis	1.026	0.695–1.514	0.898			
HBsAg status	0.871	0.592–1.283	0.486			
AQP3	2.027	1.307–3.145	**0.002**	1.955	1.256–3.044	**0.003**

Bold fonts indicate statistically significant

### Depletion of AQP3 inhibited HCC cellular proliferation and invasion

**Fig. 2:**
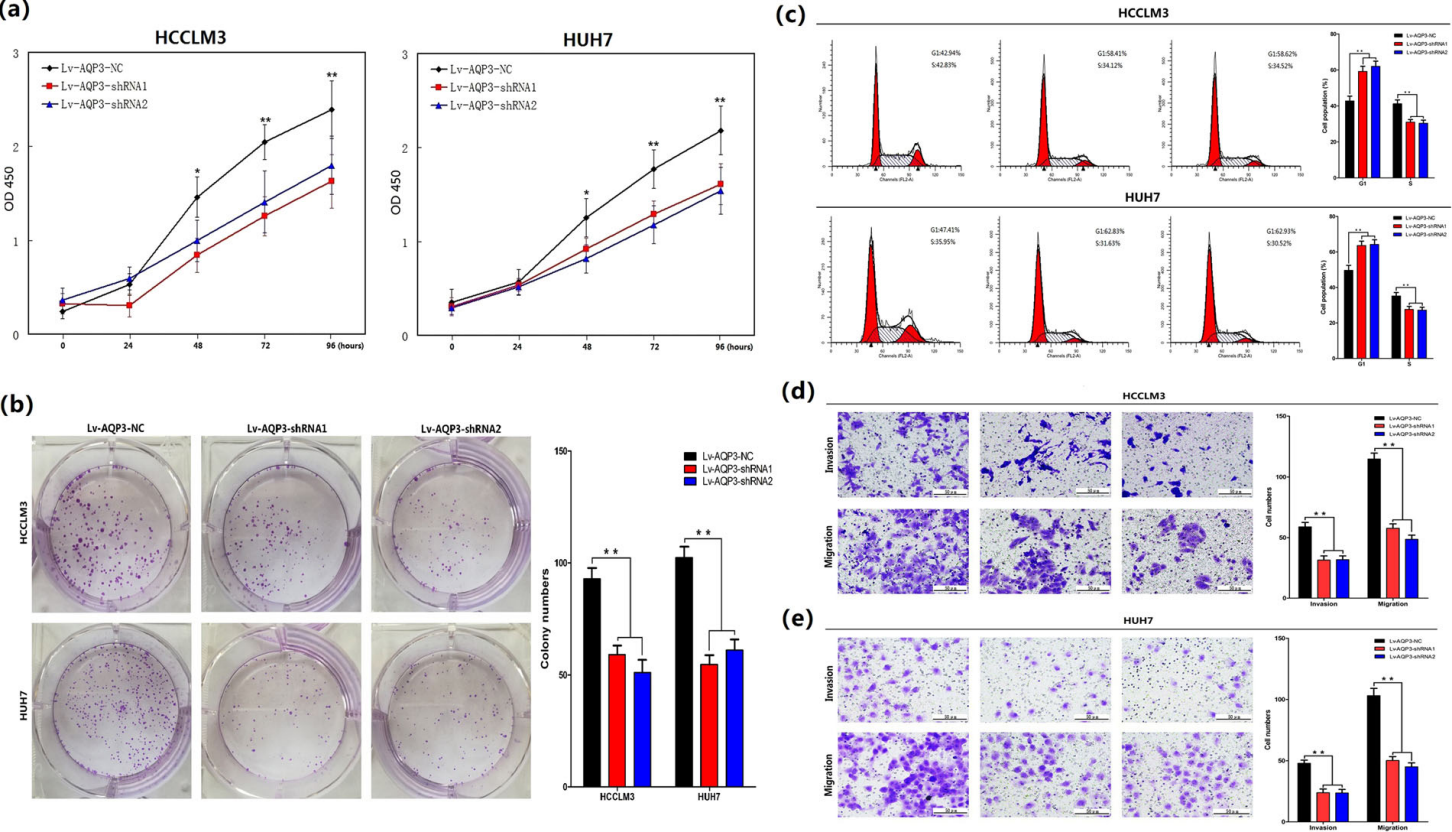
Depletion of AQP3 inhibited HCC celluar proliferration. **a** According to CCK8 assays, significant decreased proliferation rate was observed 48 h post transfection with Lv-AQP3-shRNAs compared with Lv-AQP3-NC in HCCLM3 and HUH7 cells (*P < 0.05, **P < 0.01). **b** Clonogenic assays were performed with HCCLM3 and HUH7 cells. Lower number of colonies were found in cells treated with Lv-AQP3-shRNAs compared with Lv-AQP3-NC treated cells (**P < 0.01). **c** The effect of AQP3 on the cell cycle was tested using flow cytometry analysis. In HCCLM3 and HUH7 cells, AQP3 depletion cells showed an increase in the number of cells in G1 phase and an decrease in the number of cells in S phase when compared with the Lv-AQP3-NC groups (**P < 0.01). **d**, **e** Transwell assays showed lower invasion and migration abilities were observed in HCCLM3 and HUH7 cells transfected with Lv-AQP3-shRNAs compared with Lv-AQP3-NC (**P < 0.01)

**Fig. 3:**
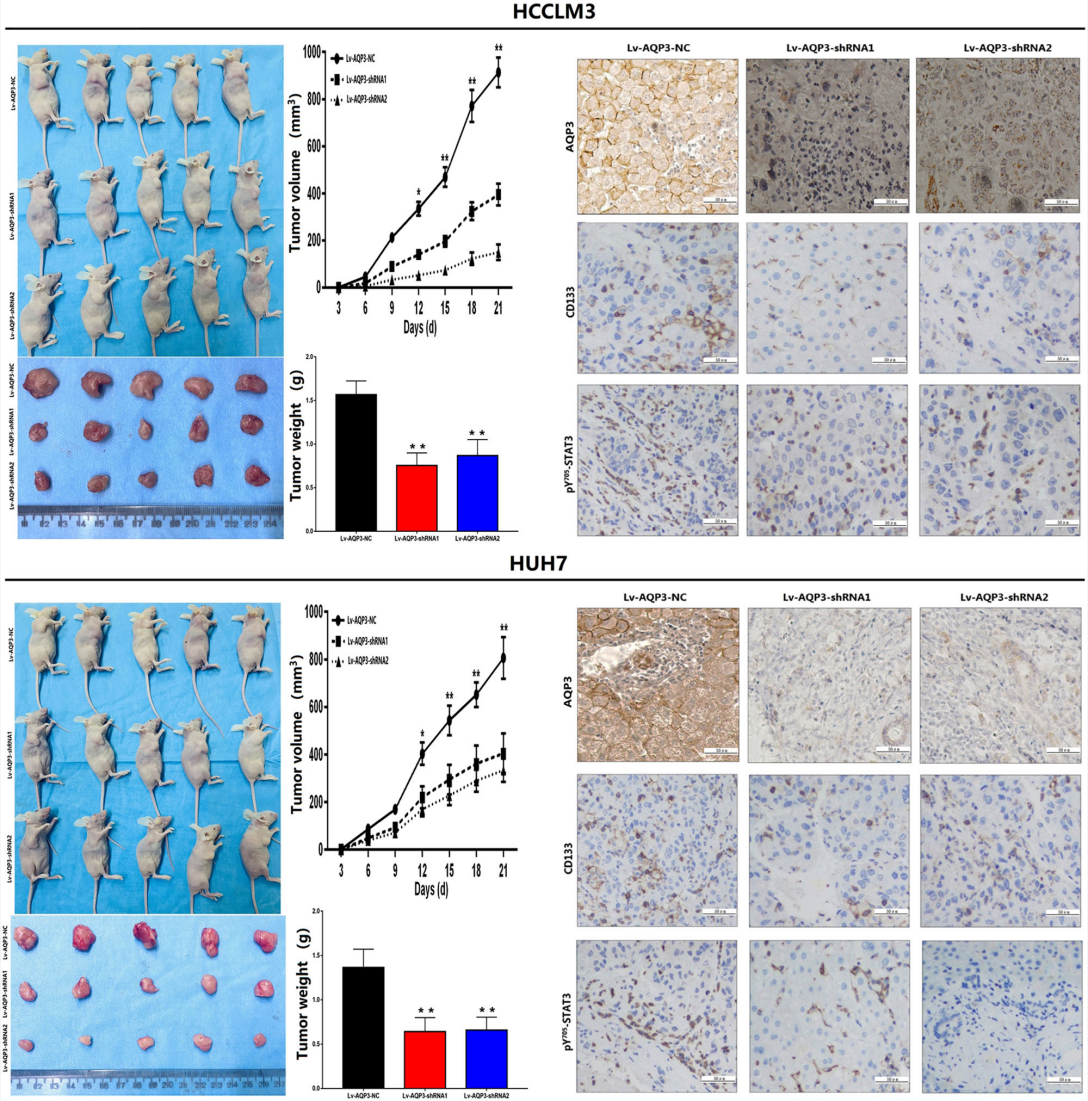
Depletion of AQP3 inhibited HCC celluar tumorigenicity. Subcutaneous tumorigenicity experiments in nude mice showed 28 days after cell injection, lower tumor weight, and volume was observed in Lv-AQP3-shRNAs groups compared with Lv-AQP3-NC groups (*P < 0.05, **P < 0.01). The subcutaneous tumors of nude mice were prepared into paraffin sections, IHC was used to test the protein levels of AQP3, CD133 and pY^705^-STAT3, results showed CD133 and pY^705^-STAT3 expression were decreased when AQP3 knockdown

Lv-AQP3-shRNAs were transfected into HCCLM3 and HUH7 cells with high AQP3 expression to investigate the roles of AQP3 on the malignant behavior of HCC cells. A significant reduction in the proliferation rate was observed at 48 h post transfection with Lv-AQP3-shRNAs as compared with that with Lv-AQP3-NC in both cell lines (*p* < 0.01, Fig. 2a). Consistent with the results of the CCK8 assay, AQP3 knockdown led to a significant decrease in the number and size of foci as compared with the Lv-AQP3-NC group (*p* < 0.01, Fig. 2b). The effect of AQP3 on the cell cycle was assessed using flow cytometry analysis. The results showed that the depletion of AQP3 increased the percentage of cells in G1 phase and decreased that in the S phase in both cell lines (*p* < 0.01, Fig. 2c). In vivo, a xenograft nude mice model was used to test the influence of AQP3 on tumorigenicity. After 21 days post cell injection, lower tumor weight and volume were observed in Lv-AQP3-shRNAs HCCLM3 and HUH7 groups as compared with the Lv-AQP3-NC groups (Fig. 3). The cell invasion and migration assays demonstrated that HCCLM3 cells transfected with Lv-AQP3-shRNA1 or Lv-AQP3-shRNA2 showed less-intense invasive and migratory capacities as compared with the control group (*p* < 0.01, Fig. 2d). The same phenomenon was also observed in HUH7 cells (*p* < 0.01, Fig. 2e).

### AQP3 expression was positively correlated with CD133

**Fig. 4:**
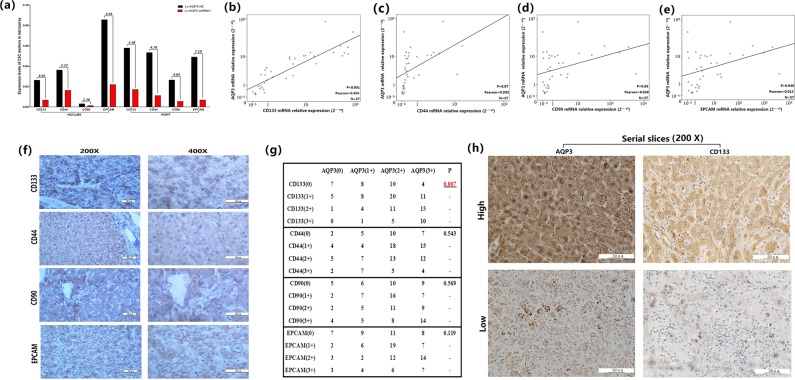
The expression of AQP3 and CD133 was positively related. **a** The Human Tumor Proliferation/Invasion RT2 Profiler™ PCR Array showed four LCSCs markers were downregulated after AQP3 knockdown in HCCLM3 and HUH7 cells. Lv-AQP3-NC/Lv-AQP3-shRNA1 was 3.91 folds (CD133), 2.22 folds (CD44), 2.26 folds (CD90), 3.91 folds (EPCAM) in HCCLM3; 3.38 folds (CD133), 4.78 folds (CD44), 4.85 folds (CD90), 7.19 folds (EPCAM) in HUH7. **b**–**e** The relationship of relative mRNA expression levels (2-ΔΔct) between AQP3 and the four LCSCs markers was calculated using Pearson Correlation Analysis. Results showed AQP3 was positive correlated with CD133 (*P* = 0.001, Pearson = 0.504). **f** The protein expression of CD133, CD44, CD90, and EPCAM were mainly located in cytomembrane according to IHC in 120 HCC paraffin section specimens. **g** The relationship of protein expression levels between AQP3 and the four LCSCs markers was calculated using chi-square test. Results showed patients with AQP3 high expression had relative higher CD133 expression compared with those with AQP3 low expression (*P* = 0.007). **h** Serial section (4 μm) in IHC showed the expression intensity of AQP3 and CD133 were positively correlated

In order to explore the mechanism of AQP3 carcinogenesis, the mRNA expression profile of Lv-AQP3-shRNA1-transfected HCCLM3 and HUH7 cells was compared with that of Lv-AQP3-NC transfected cells using a Human Tumor Proliferation/Invasion RT2 Profiler™ PCR Array containing 86 cell proliferation/metastasis-related genes (Supporting files 1, 2). Four LCSC markers (CD133, CD44, CD90, and EPCAM) were downregulated after AQP3 knockdown in both two cell lines (Fig. 4a). Next, we examined the correlation between AQP3 and the four LCSC markers in HCC specimens by qRT-PCR and IHC. The results indicated that the mRNA levels of AQP3 and CD133 were positively correlated in 37 HCC fresh tissue samples (Fig. 4b). Furthermore, a positive correlation was detected between the protein levels of AQP3 and CD133 according to IHC in 120 HCC patients. The IHC data showed that patients with high expression of AQP3 exhibited higher levels of CD133 as compared with those with low expression (*p* = 0.007, Fig. 4g, h). However, no significant correlation was observed between AQP3 and the other three LCSC markers (Fig. 4c–g).

### AQP3 regulated CD133 expression in HCC

**Fig. 5:**
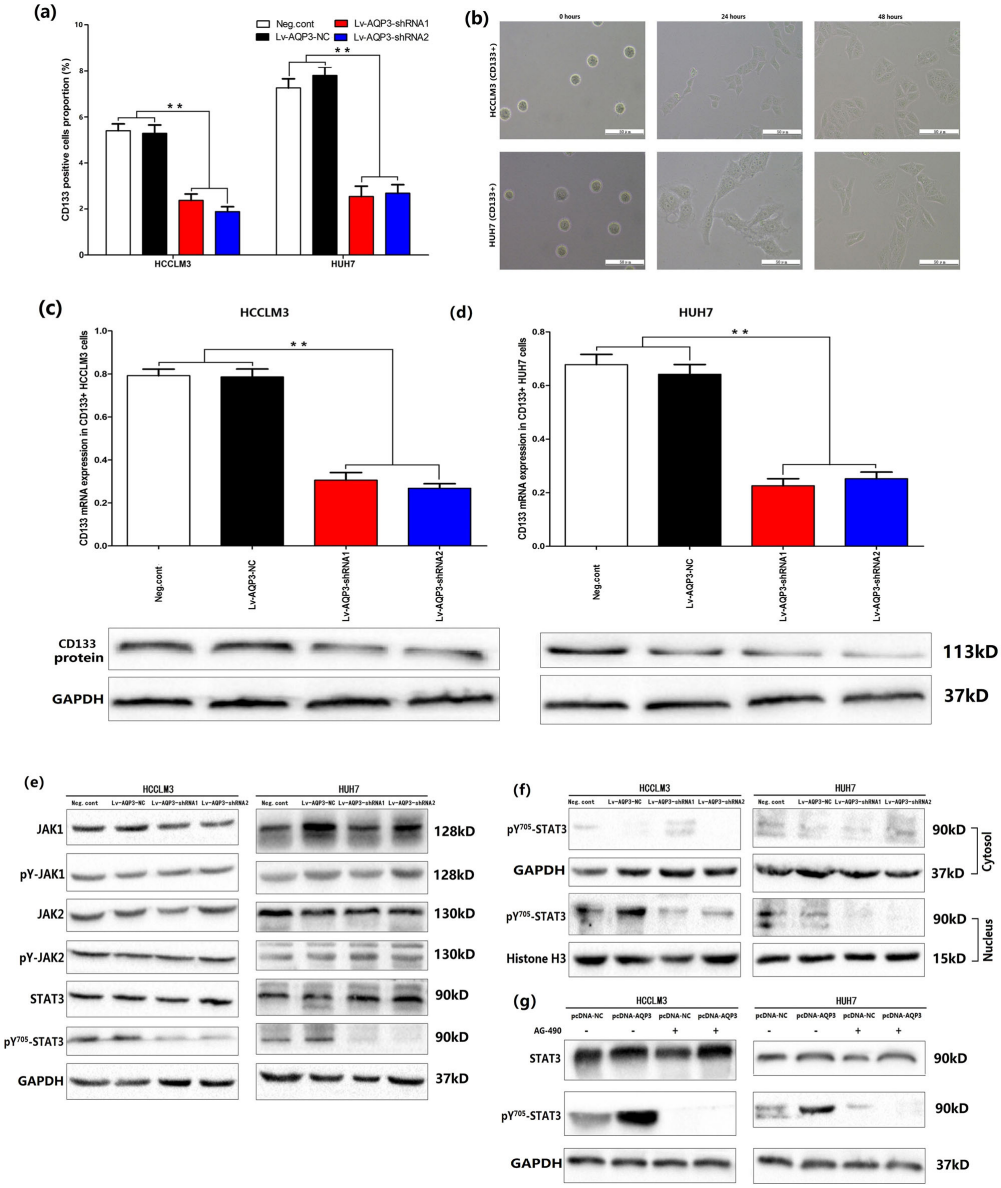
AQP3 regulated CD133 and pY^705^-STAT3 expression. **a** FACS was used to test the CD133 positive cells proportion in HCCLM3 and HUH7 cell lines. Results showed AQP3 knockdown could reduce CD133+ cells proportion (**P < 0.01). **b** The purification of CD133+ subpopulation in HCCLM3 and HUH7 cells was performed by MACS. The sorted CD133+ cells were displayed under electron microscope and cultured for 48 h into logarithmic phase to transfected Lv-AQP3-shRNAs or Lv-AQP3-NC. **c**, **d** qRT-PCR, western blotting showed AQP3 knockdown significantly inhibited CD133 mRNA and protein expression in CD133+ HCCLM3 and HUH7 cells (**P < 0.01). **e** The key protein levels in JAK/STAT3 signaling pathway was tested by western blotting in HCCLM3 and HUH7 cells. Results showed AQP3 konckdown could depress the activating STAT3 (pY^705^-STAT3) expression. **f** In HCC cells, pY^705^-STAT3 was rarely expressed in the cytoplasm and mainly expressed in the nucleus. AQP3 konckdown could significantly decrease pY^705^-STAT3 nuclear translocation; **g** pcDNA-AQP3 was used to upregulated AQP3 expression. We found AQP3 overexpression had no influence on STAT3 expression, but could promote STAT3 activation. AG-490 treatment could inhibit STAT3 activation and eliminate the influence of AQP3 overexpression

FACS was used to evaluate the proportion of CD133+ cells in HCCLM3 and HUH7 cell lines: 5.5 ± 0.7% and 7.1 ± 0.9%, respectively (Fig. 5a). Then, we transfected Lv-AQP3-shRNAs into HCCLM3 and HUH7 cells. Compared with the negative control and Lv-AQP3-NC groups, the CD133+ cell population in Lv-AQP3-shRNAs groups was significantly decreased (*p* < 0.01, Fig. 5a).

**Fig. 6:**
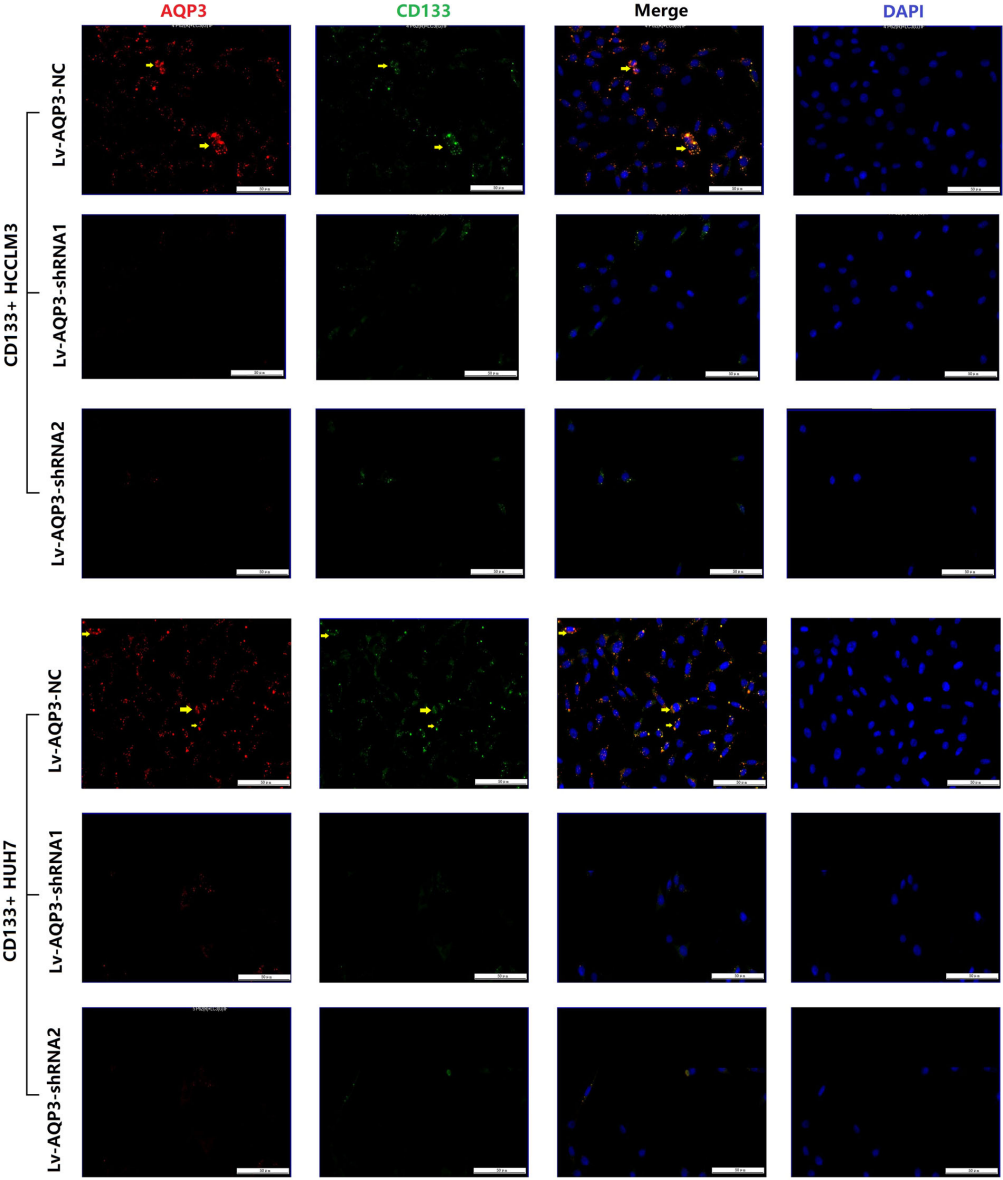
Double immunofluorescent staining assays showed AQP3 and CD133 were co-expressed and colocalized in CD133+ HCC cells. When we knocked down AQP3 by Lv-AQP3-shRNAs, the CD133 expression was also downregulated. (AQP3: red, CD133: green, yellow arrow: the colocalization of AQP3 and CD133)

The CD133+ subpopulation was purified by magnetic cell separation (MACS). Subsequently, the sorted CD133+ cells were cultured for 48 h (Fig. 5b) and transfected with Lv-AQP3-NC or Lv-AQP3-shRNAs, followed by the evaluation of the expression of CD133 mRNA and protein by qRT-PCR and Western blotting. The results showed that AQP3 knockdown suppressed the expression of CD133 mRNA and protein in CD133+ HCCLM3 and HUH7 cells (Fig. 5c, d). Double immunofluorescent staining assays were used to test the colocalization of AQP3 and CD133. Results showed AQP3 and CD133 were co-expressed and colocalized in CD133+ HCC cells. And when we knocked down AQP3 expression, the CD133 expression was also downregulated (Fig. 6).

### AQP3 maintained the stemness of CD133+ HCC cells

**Fig. 7:**
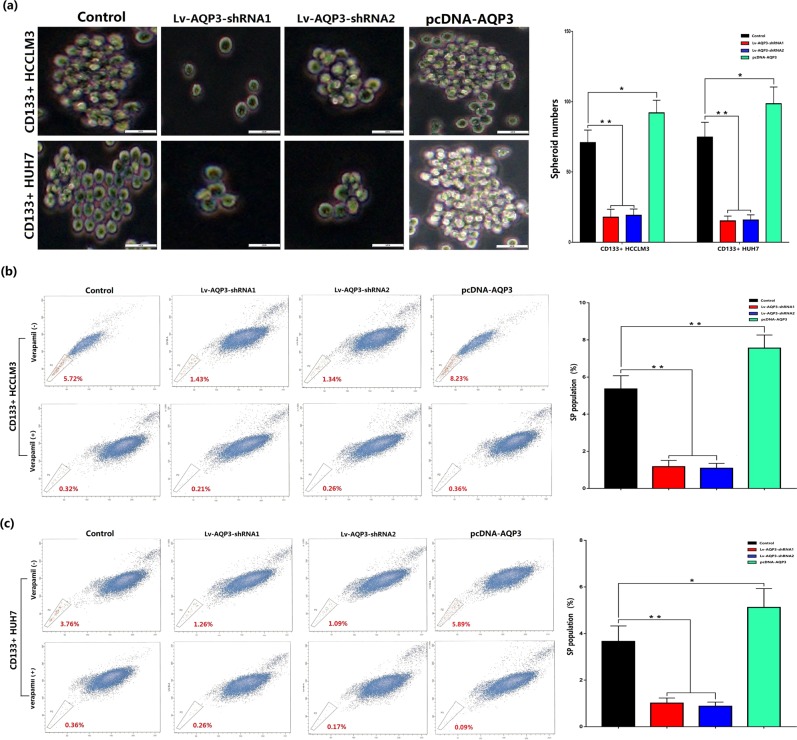
AQP3 maintained the stemness of CD133+ HCC cells. **a** The spheroid formation assay was performed to assess the effect of AQP3 on the self-renewal capacity of CD133+ HCC cells. Results showed AQP3 knockdown significantly reduced the number of spheroids (**P < 0.01) and AQP3 overexpression increased the number of spheroids (*P < 0.05). **b** The Hoechst staining and flow cytometry sorting were used to test the side population proportion in CD133+ HCCLM3 and HUH7 cells. Results showed AQP3 depletion significantly reduced the SP proportion (**P < 0.01) and AQP3 overexpression increased the SP proportion (*P < 0.05, **P < 0.01)

The spheroid formation assay was performed to assess the effect of AQP3 on the self-renewal capacity in CD133+ HCC cells. As shown in Fig. 7a, the number of spheroids formed decreased significantly when AQP3 expression in CD133+ HCCLM3 and HUH7 cells was downregulated due to Lv-AQP3-shRNAs (*p* < 0.01). The Hoechst staining and flow cytometry sorting were employed to test the side population proportion in CD133+ HCCLM3 and HUH7 cells. The results showed that AQP3 depletion reduced the SP proportion in CD133+ HCCLM3 and HUH7 cells as compared with the control groups (*p* < 0.01, Fig. 7b, c). Then, we upregulated AQP3 expression by pcDNA-AQP3 in CD133+ HCC cells. The number of spheroids and the side population proportion were both increased as compared with the control groups (*P* < 0.05, Fig. 7a–c).

### AQP3 regulated the activation of STAT3

Several studies have shown that the JAK/STAT3 signaling pathway contributed to the induction and maintenance of CSCs via the transcriptional regulation of CD133^20–22^. In order to explore the mechanism underlying the AQP3-regulated CD133 expression, we analyzed the influence of AQP3 on JAK/STAT3 signaling pathway. The results showed that AQP3 did not affect the protein expression levels of JAK1, activated JAK1 (pY-JAK1), JAK2, activated JAK2 (pY-JAK2), and STAT3 (Fig. 5e). Furthermore, AQP3 knockdown suppressed the expression of activated STAT3 (pY^705^-STAT3) (Fig. 5e) and blocked its nuclear translocation (Fig. 5f). Then, pcDNA-AQP3 was transfected to upregulate the expression of AQP3 in HCC cells. In addition, we found that pY^705^-STAT3 had higher expression levels in the pcDNA-AQP3 group than in the pcDNA-NC groups. Also, AG-490 (STAT3 activation inhibitor, MedchemExpress, USA) counteracted the active effect of AQP3 on STAT3 (Fig. 5g). In vivo experiment, we tested the CD133 and pY^705^-STAT3 expression levels in sections of nude mice subcutaneous tumors by IHC, results showed CD133 and pY^705^-STAT3 protein levels were decreased when AQP3 knockdown (Fig. 3), which was consistent with the variations in volume of subcutaneous tumors.

### AQP3 promoted CD133 transcription depending on STAT3 activation

**Fig. 8:**
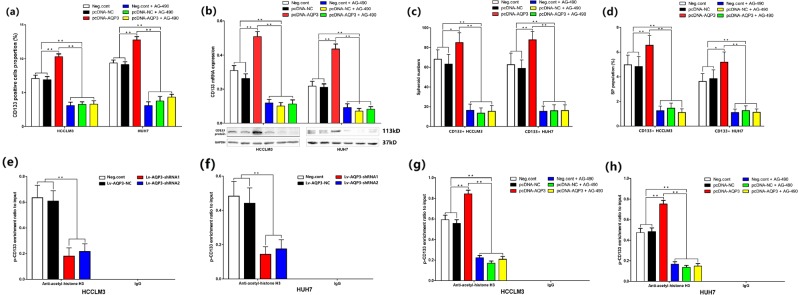
AQP3 promoted CD133 transcription depending on STAT3 activation. **a** The CD133+ cell population ratio in HCCLM3 and HUH7 cells was assessed using FACS methods. Results showed AQP3 overexpression could increase the ratio of CD133+ cell population (**P < 0.01). AG-490 could decrease the CD133 + cell proportion (**P < 0.01) and eliminate the influence of AQP3 overexpression (**P < 0.01). **b** AQP3 overexpression could up regulated CD133 mRNA and protein expression in HCC cells (**P < 0.01). AG-490 downregulated CD133 expression (**P < 0.01) and eliminated the influence of AQP3 overexpression (**P < 0.01). **c**, **d** AQP3 overexpression could enhanced the stemness of CD133 + HCC cells (*P < 0.05, **P < 0.01). AG-490 restrained the stemness of CD133 + HCC cells (**P < 0.01) and eliminated the influence of AQP3 overexpression (**P < 0.01). **e**, **f** CHIP assays was used to detect the CD133 promoter (p-CD133) acetylated histone H3 levels in HCCLM3 and HUH7 cells. Results showed AQP3 knockdown decreased p-CD133 acetylated histone H3 levels (**P < 0.01). **g**, **h** AQP3 overexpression increased p-CD133 acetylated histone H3 levels (**P < 0.01). AG-490 decreased p-CD133 acetylated histone H3 levels (**P < 0.01) and eliminated the influence of AQP3 overexpression (**P < 0.01)

Next, we upregulated the expression of AQP3 via pcDNA-AQP3 transfection to investigate the effect of AQP3 overexpression on CD133+ cell population ratio, CD133 expression, and CD133 promoter (p-CD133)-acetylated histone H3 levels in HCCLM3 and HUH7 cells. Moreover, we tested whether AG-490 inhibited the AQP3-mediated CD133 upregulation upon STAT3 activation. HCCLM3 and HUH7 cells were divided into six groups: negative control, pcDNA-NC, pcDNA-AQP3, negative control + AG-490, pcDNA-NC + AG-490, and pcDNA-AQP3 + AG-490. First, we assessed the CD133+ cell population ratio using FACS methods and found that the overexpression of AQP3 increased the ratio of CD133+ cell population in HCCLM3 and HUH7 cells (*p* < 0.01, Fig. 8a). AG-490 decreased the ratio (*P* < 0.01) and eliminated the influence of AQP3 overexpression (*p* < 0.01, Fig. 8a). Second, we tested the CD133 mRNA and protein expression using qRT-PCR and western blotting, respectively. Furthermore, we found that the overexpression of AQP3 upregulated the expression of CD133 (*p* < 0.01, Fig. 8b). AG-490 downregulated the expression of CD133 (*p* < 0.01) and eliminated the influence of AQP3 overexpression (*p* < 0.01, Fig. 8b). Finally, we detected the CD133 promoter-acetylated histone H3 levels using ChIP analysis. The results showed AQP3 knockdown decreased the CD133 promoter-acetylated histone H3 levels (*p* < 0.01, Fig. 8e, f). Moreover, the overexpression of AQP3 increased the CD133 promoter-acetylated histone H3 levels (*p* < 0.01, Fig. 8g, h), whereas AG-490 decreased the levels (*p* < 0.01) and eliminated the influence of AQP3 overexpression (*p* < 0.01, Fig. 8g, h). Furthermore, we tested the influence of AG-490 on the stemness of CD133+ HCC cells. Results showed AG-490 could not only decrease the number of spheroids and the side population proportion in CD133+ HCC cells (p < 0.01, Fig. 8c, d), but also eliminated the influence of AQP3 overexpression on the stemness of CD133+ HCC cells (*p* < 0.01, Fig. 8c, d).

## Discussion/conclusion

CD133, a widely known LCSC marker, has been proved to promote HCC proliferation and invasion^23–25^. CD133 + HCC cells possess high capacity of tumorigenicity and strong ability of self-renewal^26,27^. Accumulating evidence showed that CD133 could be used to predict and diagnose the development of HCC^28–30^. Thus, clarifying the molecular mechanism underlying the abnormal expression of CD133 during tumor progression in HCC is significant.

AQP3 is expressed in various cancer cells derived from diverse types of cancer tissues from stomach, colon, and lung^31–33^. In breast cancer, AQP3-facilitated cellular uptake of hydrogen peroxide promotes cell migration by regulating the Akt pathway^34^. Recent studies indicated a close correlation between AQP3 and stemness maintenance not only in normal stem cells but also in CSCs^11–13^.

Nevertheless, the significant role and the molecular mechanism of AQP3 in HCC progression have rarely been reported. Only one study showed that the combined overexpression of AQP3 and AQP5 in HCC might be strongly related to tumor progression and prognosis in patients with HCC^35^. However, the independent effect of AQP3 on malignant behavior of hepatoma cells and the related mechanisms are yet unknown.

In current study, we found that AQP3 was highly expressed in HCC and exerted carcinogenic roles and also delved into the mechanism underlying carcinogenesis. Thus, qRT-PCR gene array was used to screen the abnormal signal markers of AQP3 overexpression in HCC cell lines. The results showed that AQP3 expression was correlated with four LCSC markers (CD133, CD44, CD90, and EPCAM). Further experiments substantiated a positive correlation between AQP3 and CD133 expression in HCC samples, while the similar phenomenon was not observed for the other three markers. Follow-up experiments revealed that AQP3 not only regulated the CD133 + cell proportion in HCCLM3 or HUH7 cells but also stimulated the CD133 expression and promoted the stem cell-like properties in CD133 + HCCLM3 and HUH7 cells. Thus, the intrinsic molecular mechanism of AQP3-regulated CD133 expression demands further exploration.

Recent studies demonstrated that CD133 is encoded by an inducible gene, and its expression at the transcriptional level is directly regulated by interleukin-6 (IL-6)-mediated activation of STAT3^15,36^. The binding of IL-6 to its receptor gp130 causes receptor dimerization and subsequent activation of the associated JAKs. These activated JAKs, phosphorylate the receptor which now serves as the docking site for STAT3 also phosphorylated by JAKs^37–39^. The activated STAT3 translocates into the nucleus and directly binds to the promoter of CD133 leading to increased histone acetylation and subsequent transcription of CD133^15,36^.

In order to explore the mechanism of AQP3-regulated CD133 expression, we analyzed the influence of AQP3 on JAK/STAT3 signaling pathway. The results showed that AQP3 knockdown could suppress the activated STAT3 (pY^705^-STAT3) expression and blocked the pY^705^-STAT3 nuclear translocation. The AQP3 overexpression promoted STAT3 activation, and the STAT3 activation inhibitor, AG-490, counteracted the active effect of AQP3 on STAT3. Next, we investigated the effect of AQP3 overexpression on CD133 + cell population ratio, CD133 expression, and CD133 promoter (p-CD133)-acetylated histone H3 levels in HCCLM3 and HUH7 cells. The results showed the AQP3 overexpression increased CD133 + cell proportion, CD133 expression, and p-CD133-acetylated histone H3 levels. Then, we used AG-490 to validate the effect of AQP3-mediated CD133 upregulation on STAT3 activation. Thus, we concluded that AG-490 not only decreased the CD133 + cell population ratio, CD133 expression, and p-CD133-acetylated histone H3 levels in HCC cells but also eliminated the influence of AQP3 overexpression. Therefore, it could be speculated that AQP3 activated STAT3 promotes the transcription of CD133.

In summary, we clarified the carcinogenic molecular mechanism of AQP3/STAT3/CD133 signaling pathway in HCC by four pieces of experimental evidence: (a) AQP3 was overexpressed and executed oncogenic roles in HCC; (b) AQP3 upregulated the expression of CD133 and maintained the stemness of CD133 + HCC cells; (c) AQP3 accelerated the activation of STAT3; (d) AG-490 (STAT3 activation inhibitor) eliminated the promotion effects of AQP3 on CD133 transcription.

These findings indicated that AQP3 plays a major role in hepatic carcinogenesis and is closely related to the characteristics of LCSCs. This might provide a novel target or method for the detection and treatment of HCC in the future.

## Supplementary information

Supplementary Material

Supplementary figure legends

## Data Availability

All data generated or analysed during this study were included in this published article and its Supplementary Information Files.
