# Supplementary material for: Aquaporin 3 maintains the stemness of CD133+ hepatocellular carcinoma cells by activating STAT3
Source: Cell Death Dis. 2019 Jun 13;10(6):465. doi: 10.1038/s41419-019-1712-0 (PMC6565673; doi:10.1038/s41419-019-1712-0)
Supplement: Supplementary file 1 — Supplementary Material [file 41419_2019_1712_MOESM1_ESM.pdf]

Supporting files 1

Gene Name	Nucleotide Sequence
AQP3 qRT-PCR primer	sense: 5'-CCTCTGGACACTTGGATATGAT-3'
	antisense: 5'-GGGACGGGGTTGTTGTAG-3'
CD133 qRT-PCR primer	sense: 5’-ACATGAAAAGACCTGGGGG-3’
	antisense: 5’-GATCTGGTGTCCCAGCATG-3’
CD44 qRT-PCR primer	sense: 5'-CCAGATGGAGAAAGCTCTGA-3'
	antisense: 5'-GTCATACTGGGAGGTGTTGG-3'
CD90 qRT-PCR primer	sense: 5'-ACTGCCGCCATGAGAATACC-3'
	antisense: 5'-CTGGTGAAGTTGGTTCGGGA-3'
EPCAM qRT-PCR primer	sense: 5'-CTCGCGTTCGGGCTTCT-3'
	antisense: 5'-TGTAGTTTTCACAGACACATTCTTCCT-3'
GADPH qRT-PCR primer	sense: 5'-ATAGCACAGCCTGGATAGCAACGTAC-3'
	antisense: 5'-CACCTTCTACAATGAGCTGCGTGTG-3'
Lv-AQP3-shRNA1	sense: 5'-GGATATGATCAATGGCTTCTT-3'
Lv-AQP3-shRNA2	sense: 5'-CUGUAUUAUGAUUGGAUAUTT-3'
Lv-AQP3-NC	sense: 5'-GGATGAGATAACTGAAAGG-3'
CD133 promoter CHIP primer	sense: 5'-CAGTTTACACGCATTGATGA-3'
	antisense: 5'-AGCAGCAACAGGGAGCCGAG-3'

Supporting file 2

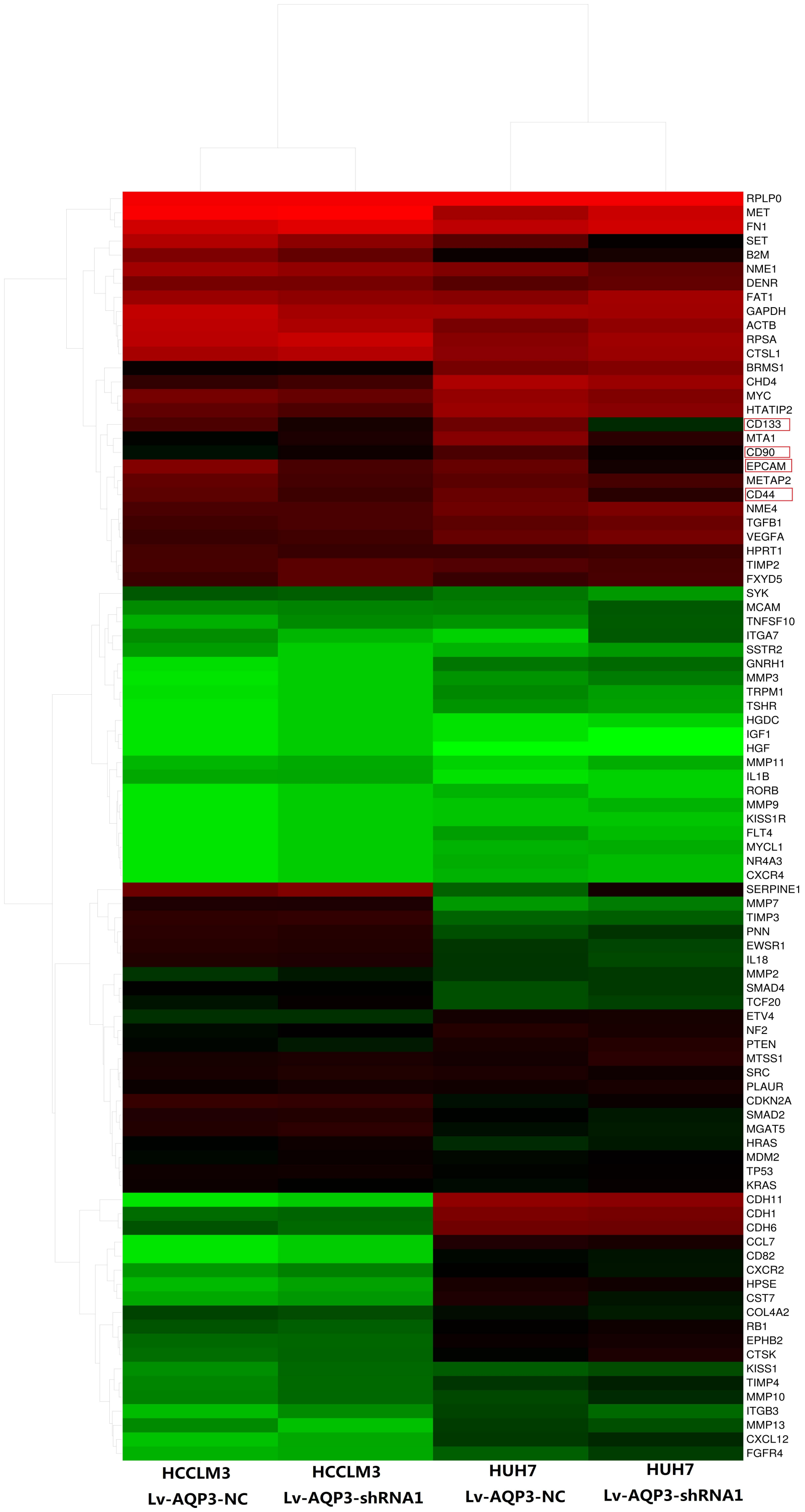

Supporting file 3

#ID	HCCLM3 NC	HCCLM3 AQP3-down	HCCLM3 Fold	HCCLM3 Comments	HUH7 NC	HUH7 AQP3-down	HUH7 Fold	HUH7 Comments
CDH6	0.00065	0.0003564	1.82491582	B	0.06287	0.054	1.1642561	B
BRMS1	0.00542	0.0055143	0.98318916	OKAY	0.068706	0.0868108	0.7914472	OKAY
CCL7	2.3E-05	0.0000363	0.62809917	C	0.010129	0.0078811	1.2852509	C
**CD44**	0.03622	0.0162921	2.22335979	OKAY	0.053291	0.01116	4.7751344	OKAY
CD82	2.3E-05	0.0000374	0.60962567	B	0.003974	0.002948	1.3478721	B
CDH1	0.00037	0.000396	0.93636364	B	0.082957	0.0648	1.2802042	B
CDH11	2.3E-05	0.0000363	0.62809917	C	0.131991	0.10512	1.2556223	C
**CD133**	0.02628	0.0067199	3.91077248	OKAY	0.057748	0.0171041	3.3762541	OKAY
CDKN2A	0.01555	0.0127578	1.21883083	OKAY	0.003295	0.0056136	0.587033	OKAY
CHD4	0.01382	0.0174273	0.79310048	OKAY	0.251479	0.14814	1.6975787	OKAY
COL4A2	0.00095	0.0006985	1.36234789	A	0.003507	0.0023844	1.4710165	A
CST7	9.1E-05	0.0001243	0.73370877	B	0.009797	0.002814	3.481588	B
**CD90**	0.00305	0.0013486	2.26190123	OKAY	0.026434	0.005448	4.8519652	OKAY
**EPCAM**	0.08556	0.0219065	3.90558054	OKAY	0.048986	0.0068136	7.1894363	OKAY
CTSK	0.00035	0.0004103	0.85400926	B	0.004354	0.0081048	0.5372223	B
CTSL1	0.19793	0.2513038	0.78759493	OKAY	0.114751	0.148628	0.7720709	OKAY
CXCL12	5.2E-05	0.0000858	0.6013986	B	0.001284	0.0017472	0.7349311	B
CXCR2	0.00013	0.0002112	0.61931818	B	0.004758	0.0028174	1.6889139	B
CXCR4	2.3E-05	0.0000363	0.62809917	C	7.73E-05	5.472E-05	1.4126181	C
DENR	0.06575	0.058619	1.12157492	OKAY	0.034353	0.0414	0.8297847	OKAY
EPHB2	0.00039	0.000385	1.02545455	B	0.006244	0.007068	0.8833553	B
ETV4	0.00149	0.0013596	1.0988526	OKAY	0.007529	0.0073788	1.0202916	OKAY
EWSR1	0.01077	0.0091465	1.17736839	OKAY	0.001359	0.0009032	1.5045182	OKAY
FAT1	0.14896	0.1049301	1.41965366	OKAY	0.103558	0.178548	0.5800012	OKAY
FGFR4	7.3E-05	0.0000836	0.87559809	B	0.00054	0.0010756	0.5018582	B
FLT4	2.3E-05	0.0000363	0.62809917	C	0.000126	5.482E-05	2.2969172	C
FN1	0.48735	0.6913577	0.70491903	OKAY	0.358598	0.4984776	0.7193866	OKAY
FXYD5	0.01678	0.0316316	0.5305454	OKAY	0.017177	0.0234283	0.733153	OKAY
GNRH1	2.6E-05	0.0000363	0.72727273	B	0.000307	0.000379	0.8113488	B
HGF	2.3E-05	0.0000363	0.62809917	C	1.22E-05	0.0000144	0.8493146	C
HPSE	6.2E-05	0.0000979	0.63738509	B	0.008249	0.0064668	1.27566	B
HRAS	0.00423	0.0056694	0.74526405	OKAY	0.001818	0.0025747	0.7061038	OKAY
HTATIP2	0.04075	0.0233167	1.74776019	OKAY	0.165914	0.1022964	1.6218994	OKAY
IGF1	2.3E-05	0.0000363	0.62809917	C	2.43E-05	1.162E-05	2.0896821	C
IL18	0.00937	0.008074	1.16106019	OKAY	0.001424	0.0008249	1.7268737	OKAY
IL1B	9.4E-05	0.000088	1.06363636	B	2.27E-05	3.815E-05	0.5941412	B
ITGA7	0.00017	0.0000616	2.82467532	B	3.36E-05	0.0005876	0.0572645	B
ITGB3	0.00006	0.0001617	0.37105751	B	0.001028	0.0003857	2.6665299	B
KISS1	0.00017	0.0003718	0.44540075	B	0.000587	0.000751	0.7810411	B
KISS1R	2.3E-05	0.0000363	0.62809917	C	5.79E-05	4.547E-05	1.2723682	C
KRAS	0.00602	0.0041217	1.45949487	OKAY	0.003769	0.0051224	0.7358404	OKAY
MCAM	0.00018	0.0002002	0.88111888	B	0.000259	0.0005876	0.4399792	B
MDM2	0.00383	0.0052899	0.72477741	OKAY	0.003902	0.0046488	0.8393977	OKAY

MET	1.35007	1.3542584	0.99690694	OKAY	0.197307	0.4410288	0.4473782	OKAY
METAP2	0.0419	0.0194711	2.15180447	OKAY	0.038649	0.0224094	1.7246884	OKAY
MGAT5	0.00977	0.0115775	0.84412006	OKAY	0.003464	0.0023841	1.4528949	OKAY
MMP10	0.00023	0.0003696	0.62337662	B	0.000939	0.0016649	0.5637326	B
MMP11	6.7E-05	0.0000825	0.81454545	B	4.03E-05	7.857E-05	0.5134598	B
MMP13	0.00019	0.0000484	3.84297521	B	0.001232	0.000735	1.6756191	B
MMP2	0.00133	0.0023353	0.56832099	OKAY	0.001456	0.0011844	1.229619	OKAY
MMP3	2.3E-05	0.0000363	0.62809917	C	0.000154	0.0002579	0.596972	C
MMP7	0.00875	0.0079629	1.09844403	OKAY	0.000138	0.0002575	0.5343447	OKAY
MMP9	2.3E-05	0.0000363	0.62809917	C	4.73E-05	7.049E-05	0.6703851	C
MTA1	0.00408	0.0073271	0.55700072	OKAY	0.102132	0.0118224	8.6388835	OKAY
MTSS1	0.00746	0.0075856	0.98317865	OKAY	0.007569	0.0118226	0.6401777	OKAY
MYC	0.06351	0.0420288	1.51102101	OKAY	0.141464	0.0822258	1.7204397	OKAY
MYCL1	2.3E-05	0.0000363	0.62809917	C	7.6E-05	8.892E-05	0.8542637	C
NF2	0.00336	0.0042669	0.78773817	OKAY	0.011176	0.0078889	1.4167013	OKAY
NME1	0.1735	0.1148246	1.51102812	OKAY	0.088911	0.0385785	2.3046872	OKAY
NME4	0.02423	0.0229955	1.053754	OKAY	0.060728	0.0759094	0.8000095	OKAY
NR4A3	2.3E-05	0.0000363	0.62809917	C	8.68E-05	5.747E-05	1.5094657	C
PLAUR	0.00546	0.0065582	0.83254552	OKAY	0.006985	0.007591	0.9201855	OKAY
PNN	0.01195	0.009339	1.27953742	OKAY	0.000796	0.0013506	0.5897082	OKAY
PTEN	0.00389	0.0022869	1.70011806	OKAY	0.00854	0.0102297	0.8348549	OKAY
RB1	0.00064	0.0004609	1.39032328	B	0.00518	0.0062974	0.8225982	B
RORB	2.3E-05	0.0000363	0.62809917	C	7.43E-05	3.104E-05	2.3949266	C
RPSA	0.31058	0.3835546	0.80973921	OKAY	0.105004	0.1630489	0.644001	OKAY
SERPINE	0.05267	0.076813	0.68564956	OKAY	0.000497	0.006759	0.0734871	OKAY
SET	0.25936	0.0992706	2.61266478	OKAY	0.03586	0.0049484	7.2467776	OKAY
SMAD2	0.00931	0.0090211	1.0321136	OKAY	0.004634	0.0025905	1.7889883	OKAY
SMAD4	0.00444	0.004037	1.0986376	OKAY	0.000788	0.001185	0.6652517	OKAY
SRC	0.00777	0.0085932	0.9047619	OKAY	0.008842	0.0062901	1.4056181	OKAY
SSTR2	0.00012	0.0000363	3.33884298	B	7.17E-05	0.0001282	0.5592614	B
SYK	0.00059	0.0004741	1.2453069	B	0.000314	0.0001351	2.3267046	B
TCF20	0.00293	0.0048004	0.60944921	OKAY	0.000792	0.0010229	0.7746619	OKAY
TGFB1	0.01955	0.0223663	0.87393981	OKAY	0.040344	0.0506147	0.7970886	OKAY
TIMP2	0.02277	0.0320727	0.70983734	OKAY	0.031435	0.0227489	1.381821	OKAY
TIMP3	0.01308	0.0127578	1.0248789	OKAY	0.000471	0.0005108	0.9223718	OKAY
TIMP4	0.00021	0.0003564	0.58249158	B	0.001438	0.0022228	0.6470266	B
TNFSF10	7.7E-05	0.0001727	0.4447018	B	0.000159	0.0005504	0.2882992	B
TP53	0.00614	0.0059103	1.0393381	OKAY	0.00439	0.0049904	0.8797277	OKAY
TRPM1	2.6E-05	0.0000363	0.72727273	B	0.000202	0.0001185	1.7069822	B
TSHR	2.3E-05	0.0000363	0.62809917	C	0.000159	0.0001023	1.5512902	C
VEGFA	0.0161	0.0173074	0.93012238	OKAY	0.04967	0.0629015	0.7896439	OKAY
ACTB	0.32377	0.1971684	1.64209072	OKAY	0.076438	0.1231049	0.6209202	OKAY
B2M	0.07818	0.0381414	2.04986707	OKAY	0.006304	0.0074825	0.8424746	OKAY
GAPDH	0.38237	0.1704593	2.24316303	OKAY	0.199307	0.1711055	1.1648199	OKAY
HPRT1	0.02154	0.0149622	1.43946746	OKAY	0.017126	0.0175047	0.9783907	OKAY
RPLP0	1	1	1	OKAY	1	1	1	OKAY
HGDC	2.3E-05	0.0000363	0.62809917		2.84E-05	3.859E-05	0.736133	
