# Supplementary material for: Aquaporin 3 maintains the stemness of CD133+ hepatocellular carcinoma cells by activating STAT3
Source: Cell Death Dis. 2019 Jun 13;10(6):465. doi: 10.1038/s41419-019-1712-0 (PMC6565673; doi:10.1038/s41419-019-1712-0)
Supplement: Supplementary file 2 — Supplementary figure legends [file 41419_2019_1712_MOESM2_ESM.docx]

**Supplementary Material**

Supporting file 1: The nucleotide sequence in current study.

Supporting file 2: The thermal map of The Human Tumor Proliferation/Invasion RT2 Profiler™ PCR Array.

Supporting file 3: The expression difference of 86 genes in The Human Tumor Proliferation/Invasion RT2 Profiler™ PCR Array.
